# Nature’s pulse power: legumes, food security and climate change

**DOI:** 10.1093/jxb/erx099

**Published:** 2017-05-11

**Authors:** Michael J. Considine, Kadambot H.M. Siddique, Christine H. Foyer

**Affiliations:** 1 The UWA Institute of Agriculture, The University of Western Australia, Australia, LB 5005, Perth WA 6001, Australia; 2 Department of Agriculture and Food Western Australia, South Perth WA 6151, Australia; 3 Centre for Plant Sciences, Faculty of Biological Sciences, University of Leeds, Leeds, LS2 9JT, UK

**Keywords:** Crop resilience, food security, genomics, grain legumes (pulse crops), legume breeding, orphan crops, RNA sequencing, symbiotic nitrogen fixation.

**Global food security requires a major re-focusing of plant sciences, crop improvement and production agronomy towards grain legumes (pulse crops) over coming decades, with intensive research and development to identify climate-resilient species and cultivars with improved grain characteristics. Labs contributing to this special issue have undertaken research and breeding to improve pulse crops, together with innovative production agronomy which contributes to the sustainability of cropping systems. The reviews and research together form an invaluable resource for the research community and policymakers.**

The value of pulses in food cultures around the world is well known, and calling them ‘little marvels’ is apt (BBC Radio 4 Food Programme, broadcast in the UK, July 2016), not least because of their significant health benefits (Foyer *et al.*, 2016) (Box 1). However, the use of legumes in agriculture and the genetic improvement of important grain legumes have lagged behind cereal crops. The Food and Agriculture Organization of the United Nations (FAO) facilitated the International Year of Pulses in 2016, focusing on the contribution of pulses to production and dietary diversity to eradicate hunger and malnutrition. This initiative was introduced with objectives to (i) promote the value and utilization of pulses throughout the food system, (ii) raise awareness of their benefits, (iii) foster enhanced research, (iv) advocate for better utilization of pulses in crop rotations, and (v) address challenges in trade. The FAO initiative was also linked to a growing recognition of the contribution of pulses to critical targets under Sustainable Development Goal 2, particularly regarding food access, malnutrition and smallholder incomes, as well as sustainable and resilient agriculture.

**Box 1.Diverse market classes within each grain legume (pulse) species**

**Figure:**
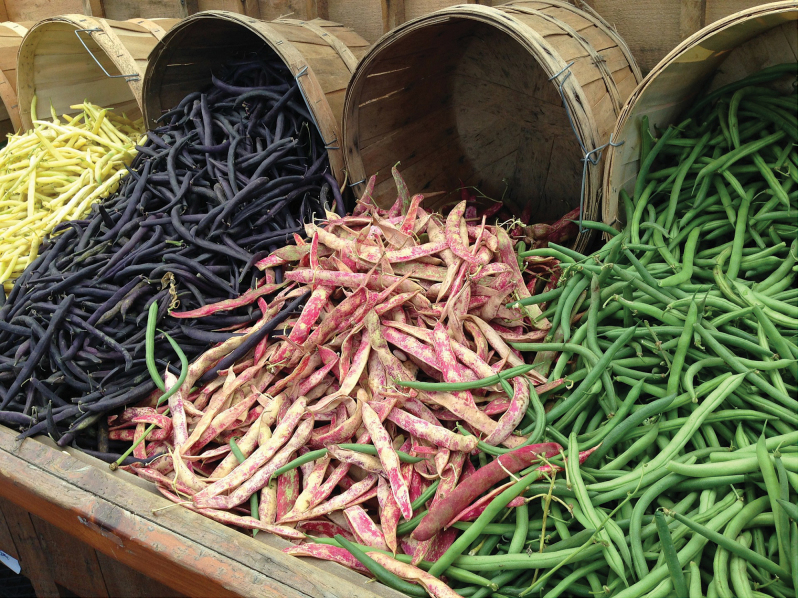
Image: Pixabay, CC0 Public Domain.

Recognizing that increasing the global production of grain legumes has the potential to provide a sustainable solution to food and protein security, significant efforts are currently being made to increase genomic resources and apply innovative breeding techniques to improve the yield and nutritional quality of legume crops, together with enhanced resilience to climate change. Production agronomy and crop rotation approaches could also be intensified to address the associated economic and environmental challenges. The papers presented in this special issue bear testimony to the urgent need for the intensification of basic and applied research into grain legumes, which will form a cornerstone of future food and nutritional security and a global web of biodiversity.

## Improved genomic resources and breeding tools

Several papers in the issue highlight the development of genetic resources that will unleash significant untapped potential for genetic improvement. The development of effective phenotyping and breeding approaches is a challenge for the less-well studied grain legumes in particular. Modern breeding efforts to improve yield, disease resistance and quality are constrained by a low level of genetic diversity in breeding programmes. Large genetic diversity exists in seeds of grain legumes held in gene banks, but these are not fully used in active breeding programmes. Cowling *et al.* (2017) explore the concept of evolving gene banks, applying optimal contribution selection to manage long‐term genetic gain and genetic diversity in pre-breeding populations. They simulated pre-breeding using a founder population based on crosses between elite crop varieties and exotic lines of field pea, subjecting the population to 30 cycles of recurrent selection for an index comprising four economically important traits. They conclude that optimal contribution selection provides the control necessary to actively improve evolving gene banks for economic traits, while maintaining high levels of genetic diversity. This revolutionary plant breeding system will allow breeders to access valuable genes that have been lost through modern breeding programmes. The plant breeding method described by Cowling *et al.* captures valuable genes from wild relatives and moves them into the breeding programme by crossing the genetically diverse exotic lines with elite lines, creating evolving gene banks. The new rapid-cycle plant breeding method will have long-term benefits for all plant breeders, and could help to adapt and develop climate-ready crops, but the immediate challenge is to validate the results in commercial pulse crops.

As one of the five major crops, soybean is the most widely planted and highest-yielding grain legume (Foyer *et al.*, 2016). The comprehensive overview of current genomic resources from functional sequences to epigenomics provided by Li *et al.* (2017) discusses the value of improving the soybean resilience to different climate change scenarios. High-throughput genomic technologies including genome sequencing, genome re-sequencing (DNA-seq) and transcriptome sequencing (RNA-seq) are being applied to a range of legumes. New insights into the giant faba bean (*Vicia faba*) genome are provided by Cooper *et al.* (2017) who used a combination of DNA-seq and RNA-seq to improve genomic resources in soybean. Using RNA-seq analysis, Du *et al.* (2017) present interesting new data on the regulatory networks that control seed set and seed size in soybean and identify hub genes that control these processes.

## Adapting to climate change through crop resilience

The genetic and biotechnology resources that are currently being applied to drought and water-logging are described by Valliyodan *et al.* (2017), within the context of existing QTLs and breeding approaches. The effects of terminal drought leaf parameters, seed set and pod abscisic acid concentrations are reported in chickpea (Pang *et al.*, 2017). Moreover, the crucial importance of root trait variability and its role in facilitating stress tolerance in chickpea is reported (Chen *et al.*, 2017). The depletion of soil water often brings the added burden of salt stress to limit plant productivity. The beneficial effects of sucrose infusion at the reproductive stage of chickpea production are presented by Khan *et al.* (2017), together with evidence that salt-stressed chickpea is carbon-limited. Hence the provision of sucrose improves vegetative and reproductive growth in plants exposed to high salt (Khan *et al.*, 2017). Another study in this issue presents novel findings showing that a drought-responsive legume, miR1514a, triggers phasiRNA formation through modulation of a NAC transcription factor (Sosa-Valencia *et al.*, 2017).

The mechanisms that underpin drought tolerance in legumes are further elaborated by an innovative analysis of root xylem plasticity and its role in improving water use efficiency in soybean plants subjected to water stress (Prince *et al.*, 2017). These and other papers in this special issue not only highlight the importance of the availability of water to legume agriculture, but also demonstrate that both drought and flooding pose some of the greatest challenges to the current and future production of soybean and forage legumes (Striker and Colmer, 2017). Their comprehensive analysis of the diversity in forage legumes for flooding tolerance will be of particular interest to those engaged in gaining a deeper understanding of the physiology of stress tolerance in legumes. It is also of practical use to researchers and agronomists engaged in forage plantings in flood-prone areas. Data for some key species are provided, in which current eco-physiological understanding is limited. Suggestions for future areas of priority in this important group of plants include the central importance of understanding anoxia tolerance in roots, the ability to maintain symbiotic nitrogen fixation during waterlogging in the field, and identification of traits conferring the ability to recover after water levels subside (Striker and Colmer, 2017).

Different aspects of reproductive physiology are described in well-considered and thought-provoking reviews by Cao *et al.* (2017) and Ozga *et al.* (2017). Cao *et al.* examine the dependence of soybean flowering and stem growth habits on day length, highlighting the interplay between photoperiod and miRNA-mediated flowering modules in soybean. Meanwhile, Ozga *et al.* cast the net beyond soybean to explore how hormones integrate high-temperature stress during reproductive development in grain legumes, from meiosis to flowering, fruit set and seed maturation. Moreover, a gene expression atlas is described for pigeon pea (Pazhamala *et al.*, 2017), together with the application of this knowledge to deduce novel information regarding the genes associated with pollen fertility and seed formation.

Some grain legume species such as faba bean and pigeon pea (*Cajanus cajan*) have outcrossing characteristics, and rely to some extent on pollination by animal vectors. Climate change and associated extreme weather events such as sudden episodes of high temperature during flowering can affect reproductive success in grain legumes, directly through physiological damage and indirectly by affecting plant–pollinator interaction. Bishop *et al.* (2017) report a substantial increase in the level of outcrossing in faba bean by insect pollinators following heat stress both in a controlled environment and under field conditions. Stoddard, in his Insight article, discusses the ability of faba bean to self-pollinate in the absence of bee activity, known as ‘autofertility’ (Stoddard, 2017). It is argued that reliance on wild pollinators is a risky strategy when also affected by climate change. Thus the provision of honey bees may be increasingly required for adequate pollination of faba bean crops in the future.

## Neglected orphan crops

As a protein staple in the diet of many of the world’s poorest, pulses are nature’s gemstones because they are protein packed and nutritious. However, in many cases relatively little is known about the biology of the plethora of ‘orphan’ legume crops that contribute to human and animal diets. Cullis and Kunert (2017) address the issue of under-utilized grain legumes directly, highlighting the considerable but neglected opportunities to advance grain legume agriculture by developing the existing germplasm. They recognize the investments of organizations such as the International Crops Research Institute for the Semi-Arid Tropics (ICRISAT), International Centre for Agriculture Research in the Dry Areas (ICARDA) and the Kirkhouse Trust, as well as initiatives to develop genomic data for these underused crops, but emphasize the need for crop physiology and production agronomy to support future crop development. Moreover, Kim and Cullis (2017) describe the chloroplast genome of marama (*Tylosema esculentum*) for the first time.

## Interactions with the soil microbiome

Many unique functions of grain legumes take place beneath the soil. These plants offer the promise of more sustainable use of nitrogen fertilizers in crop systems through their ability to fix atmospheric nitrogen in symbiotic root nodules. The process of bacteroid infection in legume roots that culminates in the formation of symbiotic nodules is summarized by Ibañez *et al*. (2017). Highlighting the differences between root-hair entry and intercellular invasion, these authors explore the evolution of this process. Nodulation, however, is suppressed in soils replete with nitrogen, for example in farms with a long history of nitrogen fertilization. This phenomenon is explored by Murray *et al.* (2017), who provide a comprehensive overview of emerging knowledge on nitrogen sensing in legumes and explore the complexity of physiological and molecular signalling and responses. With a key focus on the role of nitrate and other transporters in sensing of nitrogen availability, Murray *et al.* (2017) consider how the signalling activities of such transporters might influence nodulation.

## Crops of ancient origin come of age

The papers in this special issue describe, consider and discuss the state of our developing knowledge and understanding of important topics within legume biology, as well as identifying key areas where more knowledge is urgently needed such as an absence of comprehensive genomic information and intensive breeding efforts. In the past 50 years, global cereal production has almost tripled while pulse production has only increased by about 60% (Foyer *et al.*, 2016). The relatively low rate of yield improvement in grain legumes relative to cereals can be explained, at least in part, by the low genetic diversity in grain legume breeding programmes (Cowling *et al.*, 2017). Legume crops are also currently under-used compared to cereals in cropping systems, and yet intercropping and rotation of grain legumes with cereals or other crops have many benefits, such as enhanced crop yield, increased nitrogen-use efficiency, and reduced occurrence of plant disease. Intercropping with grain legumes is now seen as a vital mechanism for vertical intensification, as well as facilitating an increase in biodiversity and creating a more diverse landscape for animals and insects.

The importance of legumes in current and future agriculture cannot be overemphasized. Moreover, grain legumes form a minor part of current human diets, yet they are a vital source of plant-based protein and amino acids for people around the world, a versatile ingredient in human diets with a long shelf-life. The FAO recommends that they are eaten daily as part of a healthy diet to prevent and manage chronic disease, and to address growing global obesity issues (fao.org/pulses-2016). Prepare for more from these little marvels in the future.
